# Meating the moment: Challenges and opportunities for cellular agriculture to produce the foods of the future

**DOI:** 10.1038/s44319-025-00492-8

**Published:** 2025-06-06

**Authors:** Matthew J McNulty, Andrew J Stout, David L Kaplan

**Affiliations:** 1 Biomedical Engineering Department, Tufts University Center for Cellular Agriculture, Tufts University, Medford, MA USA; 2 Deco Labs Inc., Boston, MA USA

**Keywords:** Biotechnology & Synthetic Biology, Economics, Law & Politics, Stem Cells & Regenerative Medicine

## Abstract

Cellular agriculture holds great potential to usher in a modern agricultural revolution, but must first address key innovation challenges impeding cultivated meat and fish from becoming mainstream consumer products.

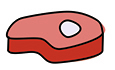

Food production systems are continually evolving along with new technologies. From the plant and animal domestication of the first agricultural revolution to the fertilizer chemistries and mechanization of the third agricultural revolution, to the advent of genetic modifications, technological advancements have fundamentally changed the way we produce, distribute, and consume food. Cellular agriculture is poised to be the next step of this continuous process of leveraging technology to improve food production efficiency and provide economic, nutritional, and social benefits.

## Cellular agriculture in response to societal demands

Cellular agriculture is an emerging production paradigm by which goods conventionally produced via animal husbandry are instead grown via cell culture. The process begins with a minimally invasive biopsy to collect stem cells from an animal, and that’s where the animal’s involvement ends. The rest of the process occurs in vitro in bioreactors or other culture systems. Cellular agriculture can be viewed as a translation of technological advances in tissue engineering and biotechnology, which have revolutionized biomedical healthcare, to the food system context.

“Cellular agriculture can be viewed as a translation of technological advances in tissue engineering and biotechnology, which have revolutionized biomedical healthcare, to the food system context.”

The social and economic benefits of the technological advances of major agricultural revolutions were realized to meet the needs of the time: the first agricultural revolution provided a reliable source of food for urban settlements with an increasing division of labor, while the third agricultural revolution increased the caloric output of the agricultural system in response to a rapidly growing human population. Cellular agriculture is similarly positioned to meet the current needs at a time when humanity demands more meat and animal products than ever while simultaneously facing finite land and water resources, crises of antimicrobial resistance, zoonotic outbreaks and climate change, and a desire for healthier, more nutritious, and more ethically produced foods.

## The rapid rise in demand for food alternatives

Consumers have been voting with their wallets to bring food alternatives—including plant-based meat analogs, milk alternatives, and dairy-free cheese—to the market. The past five years have seen a rapid rise in the appearance of these alternatives on grocery shelves, restaurant menus, and billboards. Focusing on just plant-based meat alternatives, more than 5000 product launches, as well as strategic partnerships like the Impossible Burger on fast-food menus, have signaled the promise of mainstream adoption. However, despite this momentum, consumer enthusiasm is waning and demand is plateauing due to several concerns. These include questions related to nutrition, such as relatively high sodium content, long ingredient lists fueling the ‘ultra-processed’ stigma associated with some food alternatives, differences in cooking cues and behaviors leading to sub-optimal preparations, and insufficient recapitulation of the flavors and aromas of conventional animal-derived products. It is clear that many consumers want alternatives to modern animal agriculture. It is also clear that current plant-based foods have not yet achieved mass-consumer appeal. It is our opinion that cellular agriculture can fill this gap and address the limitations of current plant-only food alternatives.

## State of the art: cellular agriculture

While the potential impact for cellular agriculture on the food production system is massive, the efforts made during the past decade have yielded little fruit—or, indeed, meat—in terms of products sold or impacts realized. This is not altogether surprising: ten years for a new technology is relatively short when considering the challenges associated with adapting expensive, low-volume biomedical techniques to inexpensive, massive-scale production of agricultural commodities. This time frame, however, is longer than the typical Venture Capital investment time horizon, and as early company predictions for market impact have come and gone, investor confidence and private funding have declined.

“… ten years for a new technology is relatively short when considering the challenges associated with adapting expensive, low-volume biomedical techniques to inexpensive, massive-scale production of agricultural commodities.”

**Figure 1:**
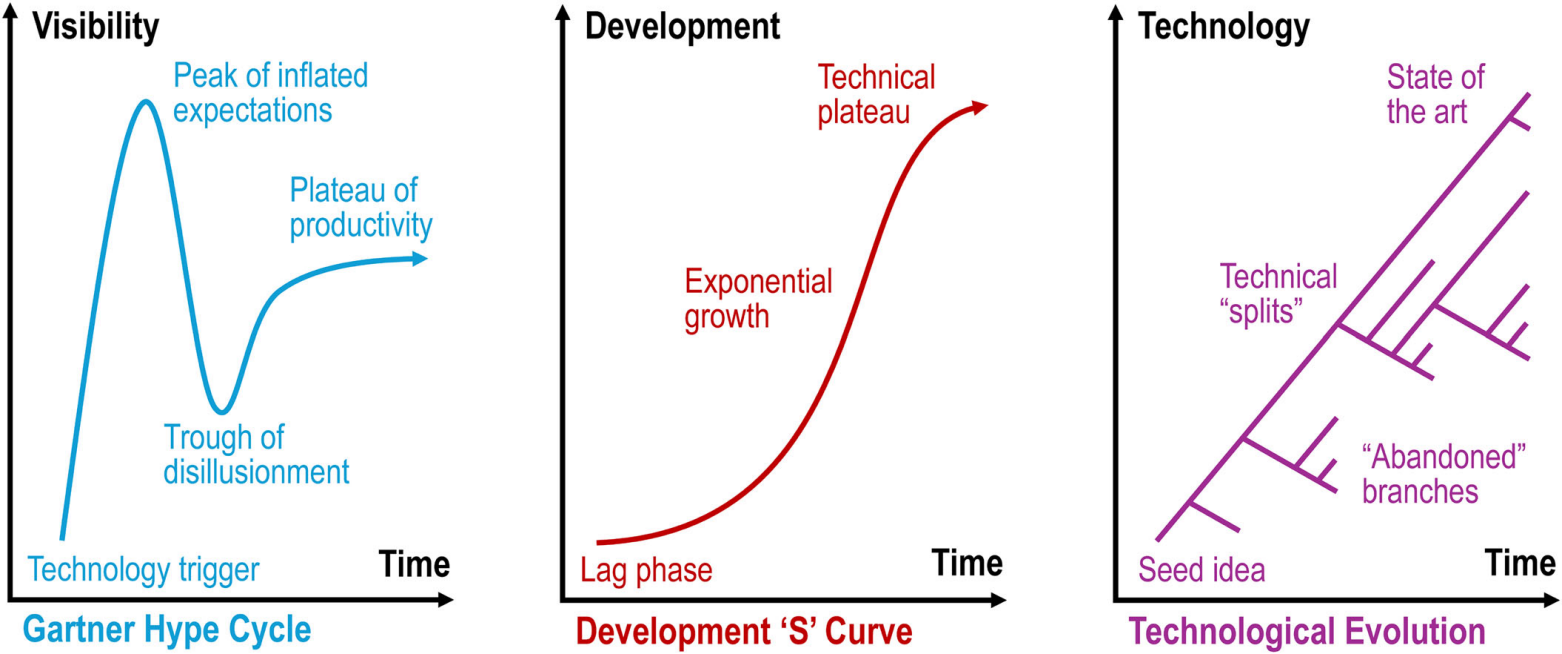
Three orthogonal models of technology evolution to interrogate the current state of cellular agriculture and next steps forward. (**A**) The Gartner hype cycle model of public sentiment around new technologies (left panel). (**B**) The ‘S’ curve model of exponential technological advancement (center panel). (**C**) The evolutionary model of technological iteration and advancement towards the state of the art for a given application (right panel).

This evolution in cellular agriculture has led proponents and detractors alike to map the technology onto various models of technical development. One of these is the Gartner Hype Cycle, a model of technological progress in which an exciting proof of concept leads to unrealistic expectations, a subsequent course-correction, and—hopefully—eventual progression towards sustainable productivity. Given waning investor confidence, it is reasonable to imagine that cellular agriculture is currently in or on the way to the “trough of disillusionment” (Fig. 1A). In our opinion, to progress to the “plateau of productivity,” a higher proportion of public resources should be deployed to develop infrastructure in academic and corporate settings, improve collaboration, support novel technologies and leverage the substantial expertise and understanding that have been developed over the last decade.

Here, it is important to remember that the Gartner Hype Cycle does not model actual technological advancement, only public sentiment. There is no “trough of dis-advancement.” Rather, development itself progresses on an “S-curve,” where new discoveries continue to advance the field exponentially until asymptotic limits are reached; at least until the next S-curve jump. Just as it is reasonable to imagine cellular agriculture in the nadir of the Gartner Hype Cycle, it is important to appreciate the field’s place on the exponential slope of this S-curve (Fig. 1B).

**Figure 2:**
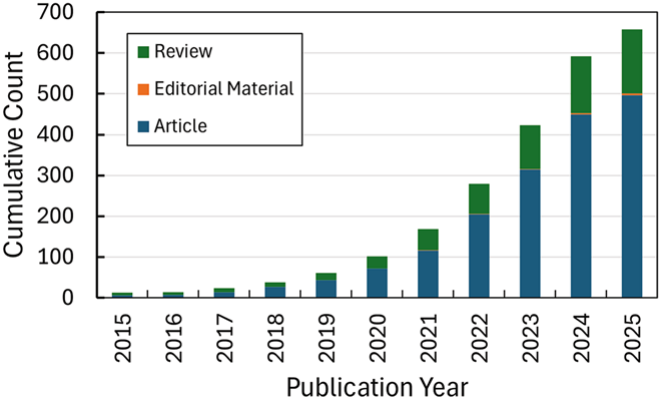
The increase of the number of publications related to cultivated meat applications of cellular agriculture from 2015. Web of Science queried on 16-Mar-2025 for keywords “cultured meat,” “cultivated meat,” “cell-based meat,” “clean meat,” “in vitro meat,” or “lab grown meat.” Note, the 2025 count is for an incomplete year representing less than one-quarter of the calendar year.

Research trends support this optimistic view. A Web of Science search for publications with the keywords “cultured meat,” “cultivated meat,” “cell-based meat,” “clean meat,” “in vitro meat,” or “lab grown meat,” retrieved only 28 articles before 2015, with all of these being either reviews or prospective assessments of consumer acceptance, environmental impacts or social implications. In 2020, total publications more than quadrupled to 125, which included thirteen primary STEM research articles with new data. By 2024, total articles quintupled to 647, including 190 articles with new data. The first quarter of 2025 has already seen 71 articles, 27 of which present new data. Although this simple search does not capture all related literature—a more comprehensive curated data base is available at the Good Food Institute—it demonstrates the rapid acceleration in publications and even faster accumulation and distribution of new data, indicating a community transition from ‘speculating’ to ‘realizing’ (Fig. 2).

Importantly, this proliferation of data sharing is happening both across scientific disciplines: cell biology, materials science, food science, molecular biology, chemical engineering, nutrition, and so on, as well as across institutional formats: academic and industry labs, collaborations or consortia between academia and industry, and infrastructure in the form of shared-access scale-up facilities or research cores. In a field that was previously disparaged for siloed and secretive company efforts and few academic resources, this has been a welcome and essential change. Much of this ethos was catalyzed by the early funders of academic research, notably nonprofit foundations such as New Harvest or the Good Food Institute, where funding was tied to open sharing of research. This paradigm established an approach that has largely propagated to support this growing field.

One of the major difficulties in building research momentum in a new field is reasonable access to starting materials and tools that are needed to establish a technology prototype. Here, that end-to-end set of materials includes relevant cell lines, food-grade media formulations, protocols for common procedures and more. Excitingly, we are now in a research environment in which many of these critical tools, materials and protocols are increasingly available, such as immortalized bovine (Stout et al, 2023a), porcine (Srila et al, 2025), and piscine (Saad et al, 2023) cells, serum-free media formulations incorporating low-cost (Stout et al, 2023b) food-grade (Charlesworth et al, 2024) components, and protocols for adapting cultures to single-cell suspension (Pasitka et al, 2023) and culturing cells in scaled-up systems (Pasitka et al, 2024). At the same time, though, many more materials and resources are still needed. These include underdeveloped cell types, such as immortalized adipogenic precursors from many species, a lack of media that offer a true path to economic viability at scale with prices under US$1/L, and a lack of integration into imminently scalable culture and food processing systems, such as scalable bioreactors or food extruders. There is a still long way to go to establish a broad and solid foundation for the field, but the trajectory is promising and can serve as a model for other emerging fields and technologies.

## Grand challenges and guiding principles for cellular agriculture

The positive impact of progress in the field is already tangible. For example, when the first cultivated beef burger was championed in 2013, its cost was estimated to be around US$300,000—or ~US$1,000,000 if allocated on a cost per pound basis. This was largely due to the use of expensive pharma-grade ingredients and lab-scale culture protocols. Fast forward 10 years, and costs are now estimated in the range of 10 s of dollars per pound to produce cultivated meat. On the lower end of this, a 2024 peer-reviewed study by authors affiliated with cultivated-meat company Believer Meats reported US$6.20/lb of cultivated chicken meat at scale when produced as a hybrid plant- and cell-based product (Pasitka et al, 2024). Along with these dramatic cost-savings, major food manufacturers have also invested significantly in start-ups and in the development of infrastructure to support food-grade, animal component-free supply chains. For instance, in 2024, Nutreco, a global corporate leader in animal nutrition, opened a dedicated facility for producing food-grade media for cultivated meat.

“Along with these dramatic cost-savings, major food manufacturers have also invested significantly in start-ups and in the development of infrastructure to support food-grade, animal component-free supply chains.”

To maintain the momentum of recent years and efficiently drive cellular agriculture research and technology into the next phase of development, we propose two challenges for cellular agriculture—namely, unlocking the “design-build-taste” cycle and developing a “Turing Test” for meat—as well as key dogmas to meet these challenges and opportunities ahead.

## Unlocking the “design-build-taste” cycle to accelerate learning

**Figure 3:**
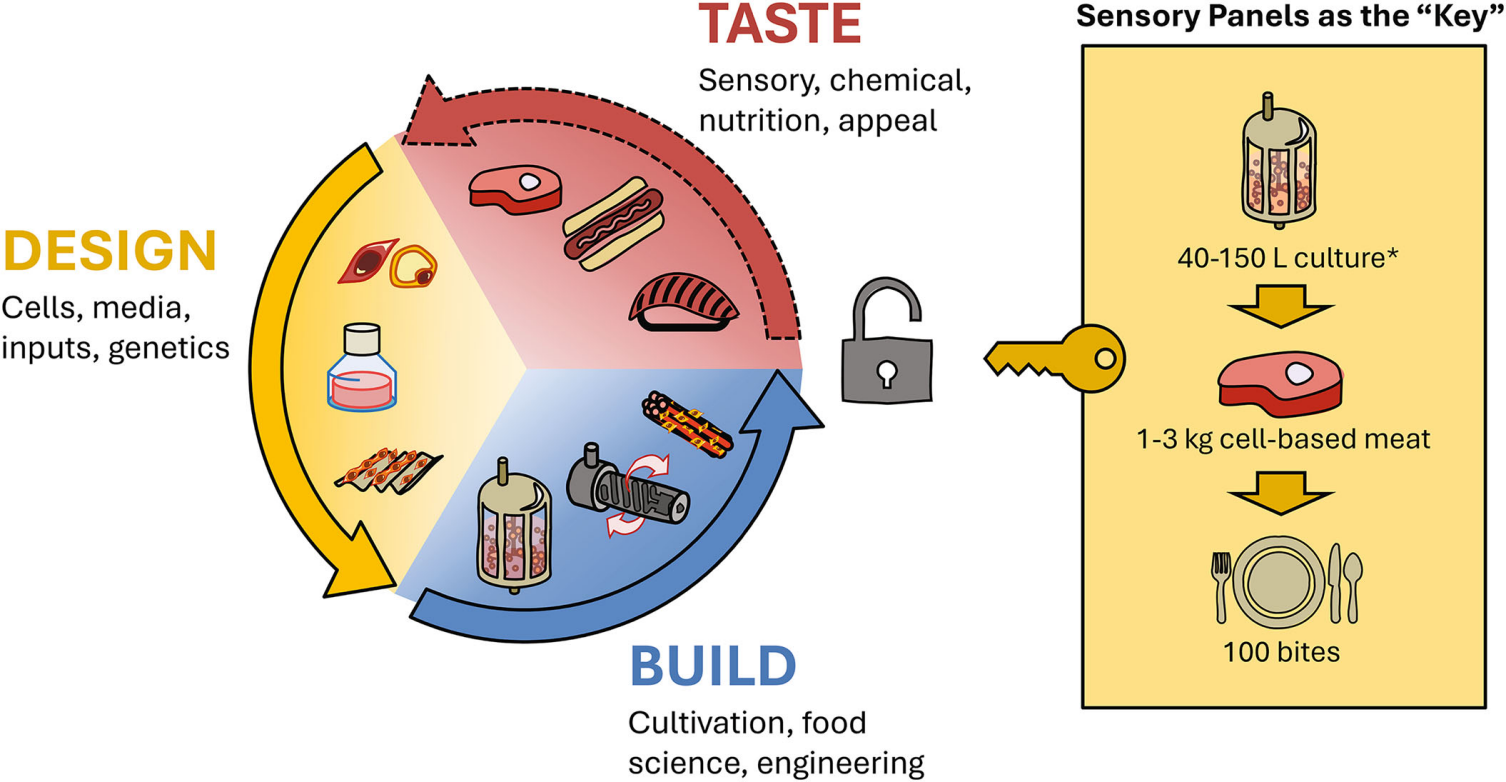
Cellular agriculture needs to unlock the “design-build-taste” cycle, which requires end-to-end expertise and cell-culture infrastructure capacities that exceed typical academic access. Culture volumes estimated to be required assume a relatively accessible stirred tank reactor performance with 3.5 × 10^−12^ kg/cell, 7 × 10^6^ cells/mL culture, and 90% recovery.

As costs continue to fall and resources continue to proliferate, the field will need to progress in both scaling *out* the number of researchers focusing on these topics and scaling *up* to produce food-relevant quantities of biomass and unlock a new “design-build-taste” cycle for cellular agriculture (Fig. 3). This cycle consists of three iterative steps: defining desired outcomes and product features; constructing a product prototype; and assessing the prototype for performance to achieve the desired outcomes.

Cellular agriculture, driven by cell culture-based biomass accumulation, must adapt to distinct engineering approaches when compared to the more traditional “design-build-test” cycle paradigm of the biopharmaceutical industry. Research and development efforts for biopharmaceuticals, which have been largely ported over to cellular agriculture, involve structuring experiments where the aim is often to generate micro- to milligram quantities of a molecule of interest. These small quantities are usually sufficient for effective and robust analytical evaluation. The insights from these evaluations can then be can then be used to guide future product and process developments, which ultimately lead to preclinical development in animals and clinical trials in humans. Cellular agriculture for food applications is challenged by the fundamental breakdown of the link between chemical assessment information and human assessment information. Understanding how, for example, the composition of what you feed cells can influence the flavor and aroma is still challenging; previously, in vivo animal tissues were not directly accessible for feeding with discrete nutrients, so this represents an entirely new whitespace to exploit in this emerging field.

“Cellular agriculture […] must adapt to distinct engineering approaches when compared to the more traditional “design-build-test” cycle paradigm of the biopharmaceutical industry.”

This means that cellular agriculture processes must generate kilograms of biomass in order to conduct proper sensory studies to evaluate—literally, “taste”—products, rather than the milligram to gram quantities for chemical assessments. Kilogram quantities of biomass for a nonoptimized production requires large culture capacities that are generally not available to most academic labs and early-stage start-ups. Using back of the envelope calculations that assume reasonable research lab performance (Allan et al, 2019), we estimate that 40–150 L culture volumes are required to produce kilograms of 100% cell-based cultivated meat (Fig. 3). Processing efficiencies to achieve higher cell densities and accessible infrastructure are both required to lower these barriers to access.

There are simpler methods to achieve this “taste” portion of the cycle, but these come with their own challenges. A lower inclusion rate of cell biomass into the final food product, for instance a hybrid meat analog with 20% cell mass and 80% plant mass, is one approach to reduce this barrier. However, this can introduce significant noise from the plant mass and cell-plant interactions, obscuring the specific functional impacts of the cellular biomass itself. There are other alternative approaches to “taste” with lower biomass requirements that can provide a portion of the feedback required for efficient learning to inform future designs, such as sensory panels for smell rather than taste, which allow for reuse of biomass across consumers due to the minimally-destructive sampling methods for smell testing (Lew et al, 2024; Zhou et al, 2025).

Unlocking the “design-build-taste” cycle will accelerate development but also provide critical insight to strengthen our understanding of the relationships between chemical, sensory and hedonic properties of food substrates. This is a new territory for biotechnology and for scaling up production systems to avoid costly paths that result in sub-optimal foods from a sensory perspective.

## A “Turing Test” for meat

Reducing the barriers to iterations of the “design-build-taste” cycle is an important step towards social and economic impact. However, perhaps equally important is the need to improve the learning outcomes from each iteration. We do not yet have sufficient expertise to perfectly reverse engineer meat: efforts to date have greatly advanced capabilities to identify appropriate raw materials and processing environments to recapitulate meat-like characteristics, but these have yet to yield an identical eating experience. As previously mentioned, the relationships between chemical, sensory, and hedonic properties of meat are not sufficiently understood to promote efficient de novo engineering of likeness from established supply chains.

The meat industry has a wealth of knowledge on how animal feed strategies, rearing conditions, slaughter methods and post-mortem treatment relate to the chemical, sensory, and hedonic properties of meat. In many ways, these insights hold limited relevance for meat from cultured cells and/or plant-based inputs, which are decoupled from the ‘whole animal’ questions and considerations. At the same time, though, they indicate how understanding and interrogating meat property relationships is critical for cellular agriculture, and can likely learn from conventional meat science. For instance, the importance of post-mortem aging in conventional meat production suggests that cultivated meat must find ways to achieve or mimic the same biochemical processes—either in a similar fashion, or through some analogous engineering. Similarly, the impact of animal feed on food functionality points towards interesting opportunities for product tuning: for instance, the possibility to tightly control media composition during cultivation in bioreactors could offer unprecedented options to modulate the content of the animal cells in terms of flavor, taste, and nutrition. In cultivated systems, this option is readily available with both non-modified as well as genetically modified cells. Further, AI and related modeling approaches will have a profound impact on these relationships, thereby impacting costs and quality outcomes for consumers, to accelerate the impact of cellular agriculture in markets in the future.

Meat is a complex and composite tissue that is created through a series of physicochemical processes—animal growth, post-mortem aging, cooling, grinding, seasoning, and heating—and associated changes. It also presents itself to consumers in a complex manner that is particularly difficult to recapitulate. This is in part because perception and preference are more closely related between taste and smell than they are in other senses; individual differences in the ability to detect certain tastes can vary a thousand-fold (Costanzo, 2024); and evaluating taste and smell is much slower than evaluating other senses due to higher dimensionality and a longer reset time needed between stimuli to minimize the impact of the previous stimulus.

**Figure 4:**
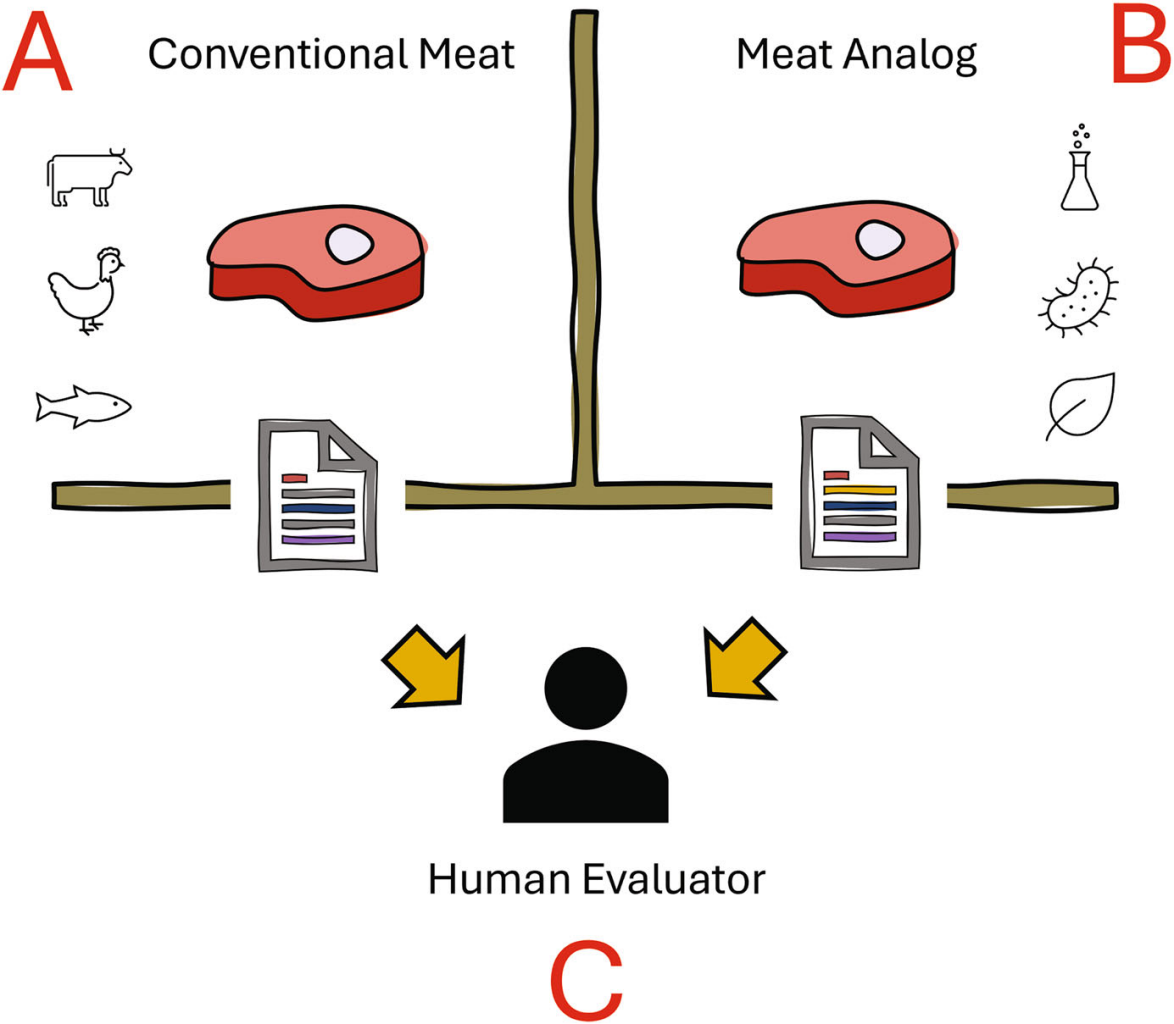
A “Turing Test” for meat that cellular agriculture and meat analogs must pass to advance the field towards efficient commercialization. In this concept, the human evaluator (**C**) is tasked with determining which protein food is a conventional meat (**A**) and which is a meat analog (**B**). They are provided only written responses to a set of questions and must rely on these responses to identify if a meat analog is sensorily and hedonically indistinguishable to real meat.

To focus scientific efforts towards societal and economic impact, a “Turing Test” for meat would be a useful endeavor. The classical Turing Test, proposed by Alan Turing in 1949, is a test of a machine’s ability to exhibit behavior equivalent to that of a human. Whereas there may be no official list of questions for the Turing Test that can most effectively interrogate humanity, the key difference in a “Turing Test” for meat would be the capacity to converge towards a minimal set of questions to predict the ability of a meat analog to exhibit sensory characteristics and elicit a hedonic experience indistinguishable from conventional meat (Fig. 4).

“To focus scientific efforts towards societal and economic impact, a “Turing Test” for meat would be a useful endeavor.”

This concept of a minimal set of questions or indicators of “meatiness” is in many ways analogous to the concepts of the quality target product profile and critical quality attributes that are fundamental to the Quality by Design (QbD) framework at the heart of product and process design of the biopharmaceutical industry. Importantly, the QbD approach establishes strong input-process-product relationships to maintain consistency and quality. However, the shape of these relationships, performance indicators and cultivated meat product attributes may ultimately assume a different shape in the distinct regulatory and consumer context of cellular agriculture. It is also worth adding that major advances in non-human assessments of sensory features for next-generation foods, such as electronic tongues and noses, may catalyze key advances for these input-process-product relationships, as will AI-related tools to empower data-intense interpretations of human preferences to enact predictions based on the analysis of ingredients, processing steps, such as cooking, or the foods themselves.

## Dogmas for the next phase of cellular agriculture

Looking forward to the next ten years of cellular agriculture, we suggest four key dogmas for R&D to meet the needs and challenges.

1. Double down on published research and distributed resources. The field must not lose sight of the fact that discovery, understanding, invention and transparent communication are vital engines for moving cellular agriculture forward, and that generating and sharing research tools is vital for enabling rapid progress, avoiding redundancy and conserving resources for innovation.
2. Integrate across technology verticals. Future foods must be realized in a collaborative way, working across cultivated meat, precision fermentation, plant-based protein, and existing agricultural infrastructure, communities and expertise. Any single vertical is insufficient on its own.
3. Consider scale at every stage. Cellular agriculture will require massive supply chain mobilization and waste management, such as feedstocks for cultivated cells, large infrastructure investment into bioreactors, storage, waste management and so on, and unprecedented biomass production to enable the “design-build-taste” cycle. All of these are required to move cellular agriculture technologies into the world of abundant commodities, and all of these should be considered at each stage of research and development.
4. Embrace creative problem solving and innovation. Simply applying biomedical and pharmaceutical R&D to cellular agriculture is unlikely to succeed, as these solutions have evolved in a medical context separate from food-relevant considerations such as food-grade versus pharma-grade reagents (Fig. 1C). Rather, cellular agriculture should explore previously unexplored or underexplored topics, probing new branches of technical development that are separate from established biomedical approaches.

## The future nostalgia of cellular agriculture in society

Cellular agriculture represents a tool for society to manifest future food systems with qualities that we hold dear: healthy, high-quality, and local nutrition for everyone. Cellular agriculture can do so by applying cutting-edge advances in tissue engineering and related biotechnology fields, with the potential to mitigate current food system difficulties of supply instabilities through recalls, seasonal variability, and animal disease, health concerns, contamination and growing food security challenges. In this way, cellular agriculture evokes a sense of future nostalgia^1^—a future of immense possibilities that taps into the values and qualities of established food systems that we love. This can revive the feeling of stopping by the local town butchery, perhaps reenvisioned as a “meat brewery”, to pick up food for a Sunday evening meal. Or this can be a means by which culturally relevant foods are reintroduced alongside the traditions and norms once associated with them, such as raw beef liver in Japan—once widely served, but banned in 2012 for health concerns.

“Cellular agriculture represents a tool for society to manifest future food systems with qualities that we hold dear: healthy, high-quality and local nutrition for everyone.”

In considering the future benefits of safety, efficiency, and social responsibility of cellular agriculture as a food system, it is important not to undervalue the potential for liberation and creative expansion. Cellular agriculture can allow agriculture, for the first time, to decouple the animal from the domestication process. No longer would animal agriculture-based food be dictated by the domestication of select animals, rather, choices can be made based on preferences towards the attributes of the food itself, or the efficacy of certain species’ cells in scaled-up cell culture systems. This could lead to freedom and opportunities to explore the largely undiscovered and diverse palette of nature’s design. Currently, about 75% of the human worldwide diet is met by 12 plant species and 5 animal species out of an estimated ~9 million living species (Wiens, 2023). This unexplored diversity in food types, tastes, and nutrition can provide new options for consumers that we have not been able to consider previously but are within the grasp of cellular agriculture. The future consumer would have an almost infinite number of food choices to foster a new generation of social benefits, human health benefits, and untold options in food formats.

It is still too early to know how cellular agriculture will enter society, or how quickly. There are many competing visions for the societal introduction of cultivated meat, including cultivated commodity meats, like chicken; cultivated premium meats, like bluefin toro; hybrid cultivated and traditional meat approaches; hybrid cultivated and plant-based meat analog approaches; wholly new and unfamiliar meat products; and pet food–early data suggests that consumers are more willing to feed cultivated meat to companion animals (Oven et al, 2022). One underexplored possible impact is ‘food as medicine’, wherein cultivated meat serves as a prophylactic approach to healthcare, given the exquisite control and tunability of food matrices and composition that cellular agriculture affords.

## Farmers, facilities, and the future

Irrespective of the ultimate shape of the product landscape, cellular agriculture will need to work hand-in-hand with farmers. The cell culture-based cultivation of meat is just a single node in the food system that will need to be embedded within a complex upstream supply chain and downstream distribution system. We view cellular agriculture not as a replacement or disruption of the current food system, but instead as an alternative, existing in parallel to other agricultural technological advances which have been taken up by farmers and integrated into their practices. Cellular agriculture will require massive agricultural inputs as raw materials for cell culture in order to operate at a food-commodity scale of production. Working with farmers will be essential for success. Cellular agriculture partnerships can provide farmers with opportunities for additional income, including monetization of otherwise low-value byproducts or cover crops, and routes to diversify their operations, allowing more options to grow crops that meet their needs of the moment, and as a result of both of these values, resilience to market volatility and crisis events.

“We view cellular agriculture not as a replacement or disruption of the current food system, but instead as an alternative, existing in parallel to other agricultural technological advances…”

Recent advances in agriculture have led to massive industry consolidation, with less than 1% of farms in the USA generating 42% of all sales (2022 Census of Agriculture data, U.S. Department of Agriculture). Much of this can be attributed to the inherent cost advantages of large-scale operation. If we value food system qualities like local and accessible production, then attention will need to be devoted to financial vehicles that can incentivize distributed production for cellular agriculture. We can look to existing efforts for inspiration. For example, the current farmers’ co-op model in agriculture to pool production resources across multiple producers who would otherwise be unduly burdened by sole ownership, could be adapted and expanded to cellular agriculture resources, for instance large-volume bioreactors. Similarly, the community solar model of financing clean energy for communities, in which customers subscribe to or own a portion of the output energy generated by a solar array, could be adapted to cellular agriculture to allow a community of consumers to buy-in to cellular agriculture ownership. Many of these more community-driven financial approaches have been largely outside of bounds for other cell culture-based biotechnologies and are thus underexplored; medical biotechnology often requires specialized training and may only serve niche populations in a community, while industrial biotechnology often operates in a business-to-business model with an emphasis on ingredients or components, rather than products. Cellular agriculture for food represents the first cell culture-based biotechnology that can provide products that are staple needs for a community. In this paradigm shift, we are hopeful for the potential of communities to drive the process in the future.

The need to provide alternatives to traditional animal-sourced goods and the promise of cellular agriculture as a manufacturing paradigm to meet that need are increasingly recognized by global leaders (United Nations Environment Programme, 2023). We have seen signs that the current food system must add alternative options or pivot to meet pending challenges in society: a projected doubling of meat consumption by 2050, food systems fueling climate change representing ~20% of all greenhouse gas emissions, looming threats of antibiotic resistance, limited land and water to meet production needs, and supply chain-disrupting disease outbreaks. For instance, 156 million poultry birds have died of H5N1 avian flu in the USA since 2022.

In the service of these needs, the cellular agriculture field has cut production prices dramatically in recent years as the scientific community has transitioned from speculating to realizing the potential of cellular agriculture. The future of cellular agriculture in society is yet to fully take form, but what is certain is that we are on our way and that the need is essential and not an option. Just as now-commonplace agricultural practices of selective livestock breeding and chemical fertilizers were once novel and unfamiliar, cellular agriculture too will take its place in our ordinary day-to-day lives—to the benefit of human, animal, and environmental health.

## Supplementary information

Peer Review File

## Footnotes

1 Credit to musical artist Dua Lipa for the creation and popularization of the term “future nostalgia”
