# Supplementary material for: Meating the moment: Challenges and opportunities for cellular agriculture to produce the foods of the future
Source: EMBO Rep. 2025 Jun 6;26(13):3229–35. doi: 10.1038/s44319-025-00492-8 (PMC12238250; doi:10.1038/s44319-025-00492-8)
Supplement: Supplementary file 1 — Peer Review File [file 44319_2025_492_MOESM1_ESM.pdf]

EMBO reports

Meating the moment: challenges and opportunities for cellular agriculture for future foods

Matthew McNulty, Andrew Stout, and David Kaplan

Corresponding author(s): David Kaplan (David.Kaplan@tufts.edu)

Review Timeline:

Submission Date:	9th May 25
Accepted:	22nd May 25

Editor: Holger Breithaupt

Transaction Report: This article was editorially reviewed.
